# How rumination influences meaning in life among Chinese high school students: the mediating effects of perceived chronic social adversity and coping style

**DOI:** 10.3389/fpubh.2023.1280961

**Published:** 2023-12-01

**Authors:** Xiaolin Yu, Jingjing Zhao

**Affiliations:** School of Education Science, Jiangsu Normal University, Xuzhou, Jiangsu, China

**Keywords:** rumination, meaning in life, perceived chronic social adversity, coping style, high school students

## Abstract

Meaning in life can be affected by many factors during adolescence. This study explored the relationship between rumination and meaning in life among high school students, as well as the mediating effect of perceived chronic social adversity and coping style. A sample of 1,275 Chinese high school students were surveyed using four questionnaires. Data analysis was conducted using Harman's single-factor test, Pearson's correlation coefficient with confidence intervals (CI), and a structural equation model. We found that rumination significantly negatively affected meaning in life among high school students (*β* = −0.28, 95% CI = −0.33–0.23). Perceived chronic social adversity (*β* = −0.14, 95% CI = −0.29–0.02) and negative coping style (*β* = −0.09, 95% CI = −0.16–0.04) each had mediating effects between rumination and meaning in life. Further, perceived chronic social adversity and coping style had chain-mediating effects between rumination and meaning in life, with both positive (*β* = −0.11, 95% CI = −0.17–0.07) and negative (*β* = −0.08, 95% CI = −0.13–0.04) coping styles showing significant effects. To enhance the meaning in life among high school students, appropriate strategies to reduce the levels of rumination and perceived chronic social adversity are needed, while also fostering appropriate coping styles.

## 1 Introduction

“Meaning in life” is the significance felt regarding the nature of one's being and existence (1). According to Erikson's theory, the adolescent stage is between the ages of 12 and 18 (2). During this period, psychological development for high school students is more intense (3), high school being a critical period to explore an individual's meaning in life and their role in the world (4). Research has found that high school students who lack meaning in life are highly susceptible to various psychological problems, such as generalized anxiety disorders, depressive symptoms, and suicidal ideation (5–8). Extant studies on adolescents at the global level have shown that from 2001 to 2020, the global prevalence of adolescent depression has increased from 24 to 37%, raising concerns about the mental health status of this population group (9). A meta-analysis of mainland Chinese high school students from 2010 to 2020 indicated that the detection rates of depression and anxiety were 28.0 and 26.3%, respectively, and showed an increasing trend over time (10). The COVID-19 pandemic has also had a significant impact on the mental health of high school students worldwide. Studies have shown that, during the pandemic, levels of depression and anxiety among children and adolescents, particularly high school students, were generally higher (11, 12). In China, during the pandemic, the prevalence rates of depression (48.72%) and anxiety (40.17%) among high school students were significantly higher than those of middle school (depression: 37.92%; anxiety: 34.09%) and college students (depression: 21.1%; anxiety: 11%) (5, 13), which may indicate an age-related effect of the higher level of psychological sensitivity or greater influence of the external environment. The current state of high school students' meaning in life in Asian countries is not optimistic. Research has found that 18% of Singaporean adolescents lack clear life goals, significantly affecting their life satisfaction, compared to 1.57% in Israel and 4.2% in the United States (14, 15). A survey on meaning in life among Chinese high school students revealed that 11.9% had never contemplated the value of their own life and more than 30% had never received any form of “meaning in life” education (16). However, a high level of meaning in life can serve as a protective factor against depression and anxiety symptoms at this age (4, 17, 18), and actively exploring life's meaning can encourage a lifelong learning orientation (19). This issue highlights the need to address the lack of meaning in life among high school students as a pressing social issue. A lack of a sense of meaning in life can lead to self-stigma (20), decreased self-control (21, 22), and a negative state characterized by anxiety and depression (4, 18), all of which pose significant threats to mental health (17, 23).

As a renowned scholar in the field of meaning in life, Steger (24) found that cognitive styles are significant sources for individuals in their pursuit of meaning in life. Rumination, or thinking obsessively about the causes and consequences of events, which can result in symptoms of negative affect, is considered a stable negative response style and a cognitive vulnerability (25). The response styles theory suggests that rumination not only affects an individual's executive functioning and problem-solving abilities (25, 26), but also increases the tendency to internalize problems such as anxiety, depression, and sleep disorders (27, 28), as well as externalizing problems such as aggression, non-suicidal self-injury, and suicide (29–31). Empirical research also indicated that rumination is an important determinant of the meaning in life (24, 32, 33). Specifically, when individuals have a higher level of rumination, their tendency to seek a fulfilling meaning in life decreases (32, 33). Moreover, rumination significantly depletes an individual's psychological resources, including hope and resilience (34, 35) and can result in a heightened sense of loneliness and hopelessness in daily life (36, 37), further reinforcing the sense of meaninglessness (33). Based on these theoretical and empirical findings, Hypothesis 1 can be put forward:

H1: Rumination significantly and negatively impacts the meaning in life among high school students.

According to the hierarchical model, the construction of meaning in life involves five processes (ranging from concrete to abstract): perceptions, actions, goals, sources of meaning, and meaning in life. The perceived meaning of social events is an important factor in the formation and development of meaning in life (38). Perceived chronic social adversity, also known as social trauma, describes the degree to which individuals perceive a series of emotionally directed stressful events that involve other individuals, including social exclusion or alienation, overcontrol, and weakness in social competition (39). On the one hand, the evaluation of long-term adverse events such as stress or bullying, is highly correlated with rumination (40). According to the response styles theory, rumination tends to lead individuals to interpret their environment negatively (25) and perceive life events as stressful (40), which in turn increases their perception of adverse events (39); in this regard, the interaction between rumination and stressful life events negatively affects mental health (41, 42). Therefore, rumination (as a cognitive vulnerability factor) exerts a detrimental influence on the mental health of high school students because of its interaction with stressful life events (30). On the other hand, a higher level of perceived chronic social adversity has been found to decrease an individual's sense of control (39), impair their interpersonal skills (43), and lead to social anxiety (44) and non-adaptive behaviors, including aggression (29). For high school students, perceiving adverse events such as social exclusion not only increases stress but also decreases academic self-efficacy and school satisfaction, ultimately leading to learned helplessness in academic performance (45, 46). With a significant decline in protective factors (21, 47–49), meaning in life for high school students is inevitably subject to considerable challenges (6). Empirical research has found that perceived chronic social adversity, such as social rejection and overcontrol, is significantly positively correlated with rumination (29, 50, 51) and significantly negatively correlated with meaning in life, demonstrating a negative impact (52, 53). Thus, Hypothesis 2 can be put forward:

H2: Chronic social adversity has a mediating effect between rumination and meaning in life.

Using the hierarchical model of meaning, the perception and encoding of stimuli (events) further activates individual behavior; thus, behavioral patterns play a crucial role in the development of meaning in life (38). Rumination may also affect high school students' meaning in life through their coping styles, which refers to generalized strategies or habitual preferences for approaching problems, with the goal of protecting themselves from negative physical or psychological consequences. These styles can be positive or negative (54, 55) in that they either protect individuals or not. Research has shown that rumination can interfere with problem-solving by generating negative emotions and cognitive patterns (25). For instance, some researchers have argued that rumination easily triggers negative cognitive appraisals among high school students, hindering the occurrence of positive behaviors and effective problem-solving, which reinforces a negative coping style (56). Meanwhile, other studies have shown that high school students with lower levels of rumination may employ positive coping styles when faced with stress (57). Overall, these results indicate a close relationship between rumination and coping styles. Furthermore, the coping styles adopted by high school students are closely related to the strength of their meaning in life (58). As individuals who adopt a positive coping style tend to exhibit higher levels of perceived meaning in life, whereas those who employ a negative coping style tend to undervalue their meaning in life (59), we posit Hypothesis 3:

H3: Coping styles have a mediating effect between rumination and meaning in life.

Rumination may affect coping styles through perceived chronic social adversity, thereby influencing meaning in life. When individuals encounter social adversity events, such as interpersonal rejection, discrimination, exclusion, and betrayal, they may react in one of three ways: by becoming prosocial, antisocial, or avoidant (60). Empirical studies have also found that, even when experiencing the same type of adversity, individual coping styles may differ because of various factors, such as personality traits (61), parenting styles (62), social support (63), and age (64). Adolescents are in a sensitive and fragile state of imbalanced physical and mental development, making them more susceptible to the impact of social adversity events than adults (64). Moreover, the degree of perceived social adversity can affect coping styles. For example, some researchers have suggested that middle school students who are less affected by adversity can solve problems by using positive coping styles such as interpersonal support and positive self-perception (63, 65). However, some researchers have noted that, when severely affected by adversity, high school students experience a significant decline in self-esteem and are more inclined to adopt a negative coping style to deal with stress (66). Therefore, a close connection exists between perceived chronic social adversity and coping styles. Accordingly, Hypothesis 4 is proposed:

H4: Perceived chronic social adversity and coping styles have a chain-mediating effect between rumination and meaning in life.

**Figure 1:**
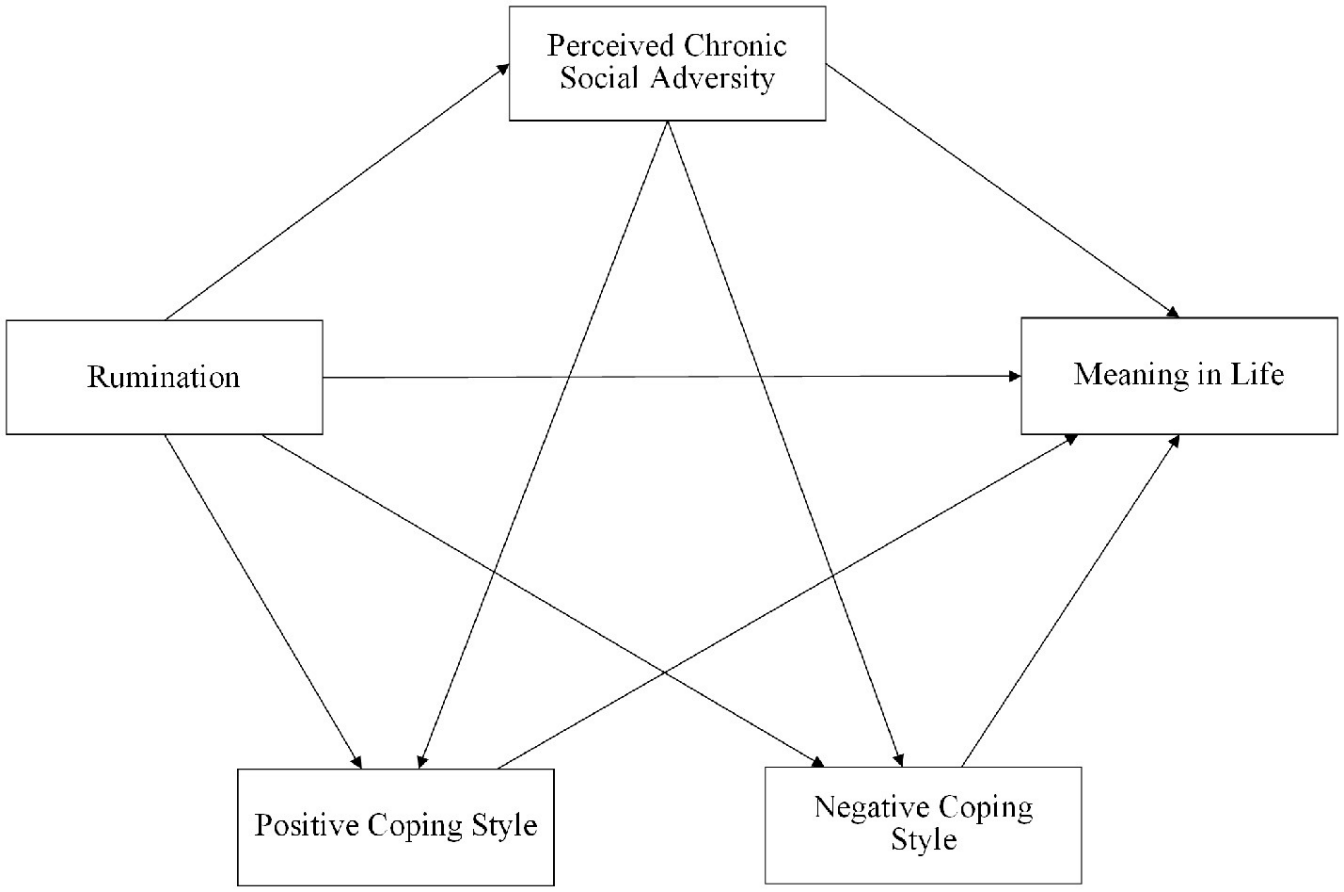
Hypothetical model of perceived chronic social adversity and coping styles between rumination and meaning in life.

As previous research on high school students' meaning in life has focused on external social and environmental factors (6, 47, 48), there is currently a lack of research on the structural relationship between cognitive factors and meaning in life. This study thus establishes a theoretical framework based on the response styles theory and the hierarchical model of meaning by proposing a chain mediation model (see Figure 1) to explore the relationship between rumination and the meaning in life felt by high school students.

## 2 Materials and methods

### 2.1 Participants and procedure

Participants were recruited from three public middle schools in Jiangsu and Guangxi provinces in mainland China using random cluster sampling. Before the formal data collection, the school leaders and students provided informed consent for this study after we explained that the study would be conducted anonymously and voluntarily, ensuring the confidentiality of participants and their right to withdraw from the survey at any time. Well-trained educators and research assistants conducted the full study protocol during regular school hours. The initial sample consisted of 1350 high school students that were 14–19 years old at the time of administering survey. Following previous studies (67, 68), after excluding participants with missing items (*n* = 4), those who had withdrawn (*n* = 12), those who did not provide consent properly (*n* = 41), as well as those who provided invariant responses for consecutive questions (*n* = 18), 1,275 valid observations remained, for an effective response rate of 94.44%. Among them, there were 661 boys (51.84%) and 614 girls (48.16%), with an average age of 16.08 ± 0.92 (see Table 1). This study was approved by the Research Ethics Committee of the School of Education Science at Jiangsu Normal University.

**Table 1:** Demographic characteristics (*n* = 1,275).

**Characteristic**	**Variable**	***N***	**%**
Gender	Male	661	51.84
	Female	614	48.16
Age	14–15	361	28.31
	16–17	815	63.92
	18–19	99	7.76
Hometown	Central of city/town	491	38.51
	Urban–rural integration zone	521	40.86
	Rural	263	20.63
Family structure	Only child	733	57.49
	Non-only child	542	42.51

### 2.2 Instruments

#### 2.2.1 Rumination

Rumination was measured using the Chinese version of the Ruminative Responses Scale, as revised by Han and Yang (69). This scale includes three dimensions (i.e., depression-related, brooding, and reflection) and 22 items (e.g., “Think about how alone you feel”). All items were scored on a four-point Likert scale (1 = never; 4 = always), with higher scores indicating more severe rumination. Extant studies have shown that this scale has good reliability and validity for Chinese samples (69). In this study, Cronbach's alpha for this scale was 0.94.

#### 2.2.2 Perceived chronic social adversity

Perceived chronic social adversity was measured using the Chinese version of the Perceived Chronic Social Adversity Questionnaire, developed by Zhang et al. (39). This questionnaire includes three dimensions (i.e., social exclusion or alienation, overly controlled, and weakness in social competition) and 28 items (e.g., “Someone arbitrarily makes most decisions for me”). All items were scored on a five-point Likert scale (1 = strongly disagree; 5 = strongly agree), with higher scores indicating higher levels of perceived chronic social adversity. Extant studies have shown that the questionnaire has good reliability and validity for Chinese samples (29). In this study, Cronbach's alpha for this questionnaire was 0.95.

#### 2.2.3 Coping style

Coping style was measured using the Simplified Coping Style Questionnaire, revised by Fang et al. (70). This questionnaire includes two dimensions (i.e., positive and negative coping styles) and consists of 20 items (e.g., “I rely on others to solve problems”). All items were scored on a four-point Likert scale (0 = rarely used; 3 = often used), with higher scores indicating a higher tendency to use that type of coping style. Extant studies have shown that this questionnaire has good reliability and validity among Chinese adolescents (70). In this study, Cronbach's alpha for the positive and negative subscales were 0.81 and 0.74, respectively.

#### 2.2.4 Meaning in life

Meaning in life was measured using the Chinese version of the Meaning in Life Scale, revised by Wang (71). This scale includes two dimensions (i.e., search for meaning and presence of meaning) and consists of 10 items (e.g., “My life has a clear sense of purpose”). All items were scored on a seven-point Likert scale (1 = absolutely untrue; 7 = absolutely true), with higher scores indicating a stronger meaning in life. Extant studies have shown that this scale has good reliability and validity among Chinese adolescents (71). In this study, Cronbach's alpha for this scale was 0.83.

### 2.3 Statistical analysis

SPSS, version 23.0 and MPLUS, version 8.3 were used for data analysis. First, we conducted Harman's single-factor test to examine common method bias in the collected data. Second, descriptive statistics and correlation analyses were performed for the main variables. Finally, we tested the chain mediation model using regression analysis in SPSS and structural equation modeling (SEM) in MPLUS. A 95% confidence interval (CI) without a zero indicated that the mediating effects were statistically significant (72).

## 3 Results

### 3.1 Common method bias

An exploratory factor analysis was conducted using Harman's single-factor test to include all variables. The results indicated that 13 factors had characteristic roots of > 1. The first factor explaining the variance was 26.61%, which is less than the critical standard of 40% (73), indicating the questionnaires used in this study had no significant common method bias.

### 3.2 Descriptive statistics and correlation analysis

The descriptive statistics and correlation coefficients among these variables are shown in Table 2. Meaning in life was significantly negatively correlated with rumination (*r* = −0.28, *p* < 0.001), perceived chronic social adversity (*r* = −0.35, *p* < 0.001), and negative coping style (*r* = −0.34, *p* < 0.001), and significantly positively correlated with positive coping style (*r* = 0.42, *p* < 0.001). Rumination was significantly positively correlated with perceived chronic social adversity (*r* = 0.69, *p* < 0.001) and negative coping style (*r* = 0.37, *p* < 0.001). Positive coping style was significantly negatively correlated with rumination (*r* = −0.27, *p* < 0.001) and perceived chronic social adversity (*r* = −0.36, *p* < 0.001).

**Table 2:** Descriptive statistics and correlations of the main variables.

**Variable**	***M***	***SD***	**1**	**2**	**3**	**4**	**5**
1. Rumination	2.18	0.66	1				
2. Meaning in life	4.89	0.94	−0.28^***^	1			
3. Perceived chronic social adversity	2.03	0.77	0.69^***^	−0.35^***^	1		
4. Positive coping style	1.91	0.49	−0.27^***^	0.42^***^	−0.36^***^	1	
5. Negative coping style	1.15	0.60	0.37^***^	−0.34^***^	0.38^***^	−0.20^***^	1

*N* = 1, 275.

*** *p* < 0.001.

### 3.3 The mediation of perceived chronic social adversity and coping styles in the relationship between rumination and meaning in life

**Figure 2:**
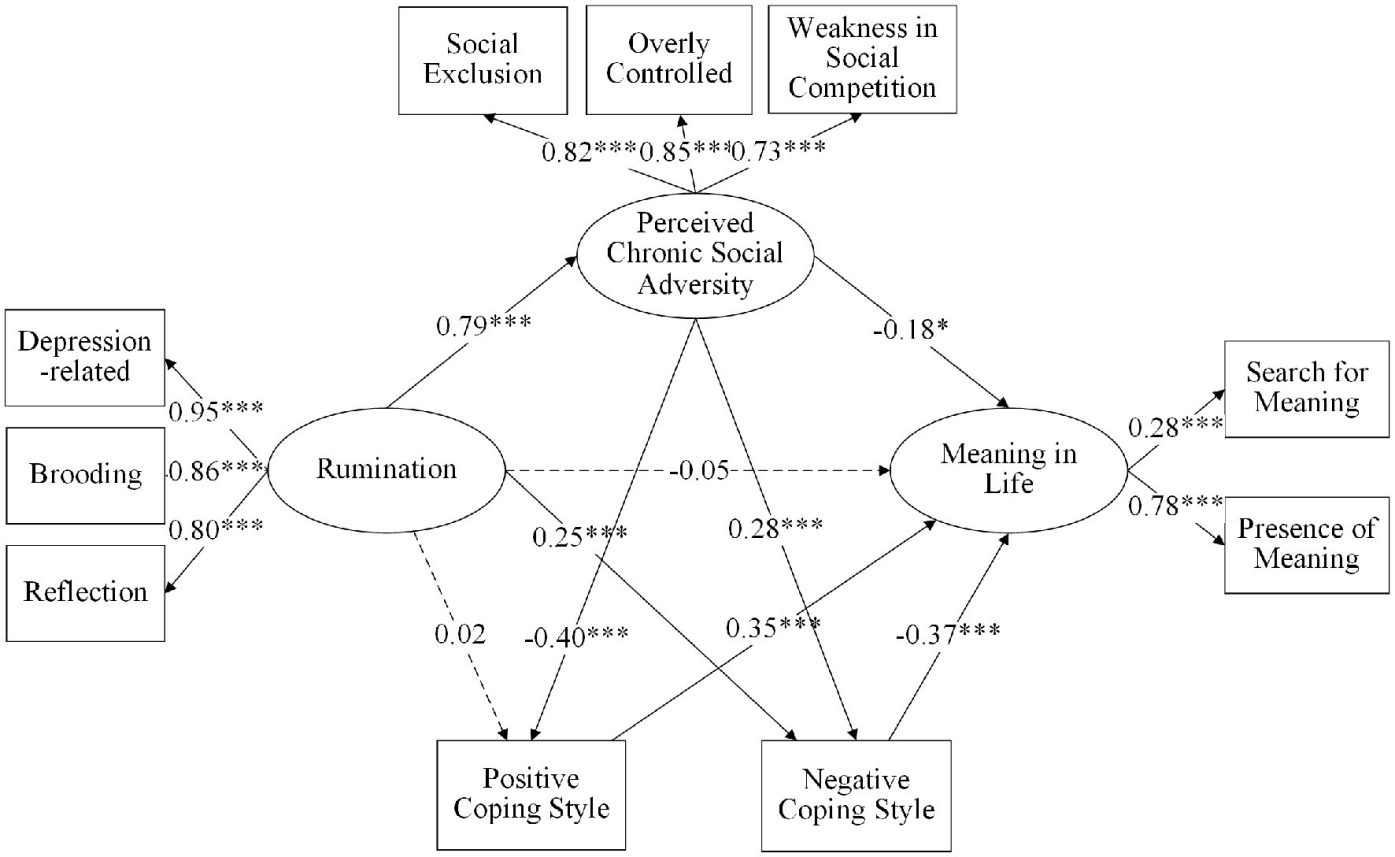
Chain mediating model of rumination and meaning in life between perceived chronic social adversity and coping styles. *p < 0.05, ***p < 0.001.

First, the regression analysis regarding the direct relationship between rumination and meaning in life revealed a significant negative impact of rumination on meaning in life (*β* = −0.28, *p* < 0.001). Second, the SEM results indicated a chain mediation effect of perceived chronic social adversity and coping styles. The model fit indices suggested that the data fit the psychometric requirements well, with χ^2^/df = 8.44, RMSEA = 0.08, CFI = 0.91, TLI = 0.89, and SRMR= 0.05. As shown in Figure 2, after introducing the mediating variables, all paths were significant, except the pathways for “rumination → meaning in life” (*β* = −0.05, *p* > 0.05) and “rumination → positive coping style” (*β* = 0.02, *p* > 0.05). Specifically, rumination positively affected perceived chronic social adversity (*β* = 0.79, *p* < 0.001) and negative coping style (*β* = 0.25, *p* < 0.001). Perceived chronic social adversity negatively affected meaning in life (*β* = −0.18, *p* < 0.05) and positive coping style (*β* = −0.40, *p* < 0.001), and positively affected negative coping style (*β* = 0.28, *p* < 0.001). Positive coping style positively affected meaning in life (*β* = 0.35, *p* < 0.001), while negative coping style negatively affected meaning in life (*β* = −0.37, *p* < 0.001).

The bias-corrected percentile bootstrap method (sample size: 5000) was used to assess the mediation effect further. The results showed that the direct relationship between rumination and meaning in life was not significant (*p* > 0.05, 95% CI = −0.20–0.12). However, as shown in Table 3, perceived chronic social adversity (indirect effect = −0.14, 95% CI = −0.29–−0.02) and negative coping style (indirect effect = −0.09, 95% CI = −0.16–−0.04) mediated the relationship between rumination and meaning in life, accounting for 29.79% and 19.15% of the total effect, respectively. Furthermore, perceived chronic social adversity and coping styles (indirect effect of positive coping style = −0.11, 95% CI = −0.17–−0.07; indirect effect of negative coping style = −0.08, 95% CI = −0.13–−0.04) played a chain mediation role in this relationship with effect sizes of 23.40% (positive coping style) and 17.02% (negative coping style) of the total effect.

**Table 3:** Mediation effect and effect size.

**Path**	**Effect**	**Boot SE**	**Proportion of total (%)**	**BootLLCI**	**BootULCI**
RUM → PCSA → MIL	−0.14	0.07	29.79%	−0.29	−0.02
RUM → NCS → MIL	−0.09	0.03	19.15%	−0.16	−0.04
RUM → PCSA → PCS → MIL	−0.11	0.03	23.40%	−0.17	−0.07
RUM → PCSA → NCS → MIL	−0.08	0.02	17.02%	−0.13	−0.04
Total mediating effect	−0.42	0.08	89.36%	−0.59	−0.29
Direct effect	−0.05	0.08	10.64%	−0.20	0.12
Total effect	−0.47	0.05		−0.57	−0.39

RUM, rumination; PCSA, perceived chronic social adversity; MIL, meaning in life; NCS, negative coping style; PCS, positive coping style.

## 4 Discussion

Based on the response styles theory and the hierarchical model of meaning, this study examined the role of perceived chronic social adversity and coping style in the relationship between rumination and meaning in life. The results indicated that rumination can directly influence the strength of meaning in life among high school students and indirectly impacts the strength of meaning in life through perceived chronic social adversity and coping style.

Rumination had a significant negative impact on meaning in life among high school students. This supports Hypothesis 1 and aligns with the response styles theory, which suggests that a key characteristic of rumination is an individual's focus on negative emotional states; such focus can easily lead to negative interpretations of the self and future events (25). Researchers argued that prolonged exposure to this negative state significantly diminishes an individual's meaning in life (32, 74–76). Specifically, humans can recognize and construct expected relationships between subjects and objects, and through reflection, discover thoughts that contradict their own meaning systems (77). When individuals are unable to resolve the discrepancy between their current and desired state, they are prone to developing a depressive self-focusing style (74). Individuals who remain in this state for an extended period will focus more significantly on their negative state, thereby weakening their inclination to seek meaning in life (75, 76).

### 4.1 The mediating role of perceived chronic social adversity

This study found that perceived chronic social adversity played a mediating role in the relationship between rumination and meaning in life, supporting Hypothesis 2. In other words, meaning in life is influenced directly by rumination and indirectly by perceived chronic social adversity. High school students are in a critical period of self-consciousness development, meaning their increasingly rich and subjectively biased personalities (3) make them highly susceptible to the negative influences of rumination and perceived chronic social adversity. This aligns with Robinson et al.'s (78) findings, who observed higher levels of rumination among students prone to negative interpretations, which intensifies their negative perceptions of the environment. Nevertheless, this dual vulnerability can impair social adaptability (79) and social problem-solving abilities (80), thereby intensifying the risk of high school students encountering social adversities such as exclusion, rejection, and control, ultimately perpetuating a destructive cycle (29). Satici (45) found that rejection can pose challenges for adolescents' self-regulation, with negative impacts on their psychological wellbeing, stress levels, and feelings of happiness. Zhang et al. (39) highlighted that rumination significantly enhances the perceived level of adversity, and can result in a sense of helplessness and loss of life goals due to prolonged exposure to adversities that ultimately weaken their perceived meaning in life (52). These results suggest that, in response to the widespread lack of meaning in life among high school students, cognitive training (such as mindfulness and positive focus) need to be strengthened during the educational process (81) to alleviate or prevent the dual negative effects of rumination and perceived chronic social adversity on the meaning in life of high school students.

### 4.2 The mediating role of coping styles

This study also found that coping styles played a mediating role between rumination and meaning in life, supporting Hypothesis 3. This suggests that rumination can further influence the meaning in life by influencing coping styles: rumination only had a significant positive impact on negative coping styles, while it did not exhibit a significant impact on positive coping styles. This is similar to the findings of Heiman et al. (82), who suggested that adolescents tend to adopt negative coping strategies after experiencing stressful events. Individuals susceptible to rumination as a negative coping style can immerse themselves in negative stimuli, which impairs their motivation and problem-solving skills significantly (25) and significantly depletes their psychological resources (83), making it even more difficult for them to adopt constructive strategies to handle these issues (26). Consequently, they can become trapped in a vicious cycle that significantly diminishes their meaning in life. Interestingly, the impact of negative coping styles is not entirely negative. For instance, many teenagers perceive that, when they choose to ignore rather than actively address individuals or events that attack them, the incidence of undesirable behaviors decreases on its own (82). However, unlike negative coping styles, positive coping styles may act as protective factors for individuals with high rumination tendencies. Loyd et al. (84) confirmed this belief, pointing out that high school students who cope with discrimination through positive thinking can help reduce the negative psychological symptoms caused by negative stereotypes and societal prejudice, thereby protecting their mental health. Therefore, if high school students adopt a positive coping style, they can resist the negative impact of rumination by maintaining good interpersonal relationships (85) and social support (63), which may further protect their meaning in life against rumination.

### 4.3 The chain-mediating effect of perceived chronic social adversity and coping styles

Our study also revealed that perceived chronic social adversity and coping styles act as chain mediators in the relationship between rumination and meaning in life, thus supporting Hypothesis 4 and the hierarchical model of meaning (38). Rumination significantly increased high school students' perceived chronic social adversity; however, the specific impact on their meaning in life varied depending on the coping styles employed by individuals, consistent with the findings by Folkman et al. (54). The diversity and complexity of coping styles adopted by individuals during social adversity events have also been reported by experimental studies (54). Similarly, Richman et al. (60) indicated that, when individuals perceive a greater likelihood of mending interpersonal relationships after social rejection, they are more inclined to adopt a positive coping style, such as engaging in prosocial behaviors, to facilitate relationship restoration. By contrast, if individuals subjectively perceive a low possibility of repairing interpersonal relationships, they are more likely to resort to negative coping styles, such as antisocial behaviors, to avoid further emotional harm (60). As a result, the different ways in which high school students cope with social adversity have varying impacts on their psychological and behavioral outcomes. This indicates that coping style choice is crucial for the development of meaning in life for high school students. However, positive coping styles are not always applicable in all situations. For example, Clarke et al. (86) suggested that adolescents who attempt to actively cope with uncontrollable stressors, such as interpersonal conflicts or illness, may exhibit more severe problematic behaviors. Therefore, the controllability of the event or environment must also be considered when employing positive coping styles. Considering the rapid development and increasing dominance of dialectical logical thinking in high school students (3), it is important to cultivate their ability to approach problems with a dialectical perspective in their daily lives. This will help them efficiently address stress and difficulties, thus alleviating the negative impact that inappropriate coping styles may have on their meaning in life.

### 4.4 Strengths and limitations

This study identified the mediating role of perceived chronic social adversity and coping styles and how these affect the impact of rumination on meaning in life within high school students. The theoretical strengths of our research include a deeper understanding of the mechanisms underlying the relationship between rumination and meaning in life. The practical strengths include new perspectives from both the cognitive and coping strategy dimensions, which can be used to improve meaning in life for these students (e.g., cognitive behavior therapy, mindfulness, positive attention bias modification, group counseling, and health education training). Due to globalization, culture mixing has become a common phenomenon in people's lives. Some researchers found that, starting from childhood, Chinese individuals typically embrace three cultures (Chinese traditional culture, western modern culture, and Marxist culture) and develop multiple identities, known as “ternary-cultural persons” (87). This multicultural influence is especially apparent among adolescents. Therefore, when exploring the current state of meaning in life among high school students, it is important to consider employing multicultural counseling strategies. Drawing from established psychological counseling theories and techniques, these strategies integrate cultural cues and individual differences (88), thus reshaping the cultural framework of mental healthcare and providing more precise assistance through available psychological health services. Additionally, previous findings emphasized the dual nature of coping styles (82, 86), which can effectively reduce stress, anxiety, and depression symptoms caused by sudden public health events (89). This perspective applies not only to local high school students but also to university students, adults, and international populations (84, 89, 90).

Despite these strengths, this study has some limitations as follows. First, the definition of rumination we used was derived from Nolen-Hoeksema (25), while recent research has further subdivided rumination into trait rumination, deliberate rumination, anger rumination, and intrusive rumination, which may have different effects on mental health (57, 91–93). Therefore, future research should further investigate the differential impacts of rumination subtypes on meaning in life. For example, deliberate rumination can, to some extent, prompt individuals to seek meaning in life and may have positive effects on mental health (92), while intrusive rumination can lead individuals to cope with stress in a negative way and thus has a negative impact on their own mental health (57). Second, while this study explores cognitive factors, interpersonal factors among adolescents may also affect meaning in life. The main source of social support for adolescents comes from their peers. However, when individuals repeatedly confide in their peers about the pressures and problems they face, they can receive negative social support if both parties focus on emotions and pressures instead of attempting to solve the problems (94). Interpersonal interaction between peers has also become more convenient with the development of the Internet, but this type of interaction may have its own unique or negative impacts (95). Hence, future research should consider other relevant interpersonal variables when exploring the relationship between rumination and meaning in life. Third, based on a comprehensive literature review and cross-sectional data analysis, this study developed a chain-mediated model to examine the impact of rumination on high school students' meaning in life, but cross-sectional studies cannot explain causal relationships. Thus, the significance of these effects requires further validation through repeated measurements and intervention studies.

## 5 Conclusions

This study investigated the relationship between rumination and meaning in life, as well as the mediating role of perceived chronic social adversity and coping styles. The results showed that rumination can directly influence the meaning in life and indirectly affect this meaning through perceived chronic social adversity and coping styles. Therefore, intervention strategies, such as cognitive behavior therapy, multicultural counseling, and fostering appropriate coping styles, should be implemented to enhance the meaning of life of high school students.

## Data availability statement

The original contributions presented in the study are included in the article/supplementary material, further inquiries can be directed to the corresponding author.

## Ethics statement

The studies involving humans were approved by the Research Ethics Committee of the School of Education Science at Jiangsu Normal University. The studies were conducted in accordance with the local legislation and institutional requirements. Written informed consent for participation in this study was provided by the participants' legal guardians/next of kin.

## Author contributions

XY: Conceptualization, Formal analysis, Funding acquisition, Methodology, Project administration, Resources, Supervision, Writing—original draft, Writing—review & editing. JZ: Data curation, Formal analysis, Investigation, Software, Validation, Visualization, Writing—original draft.
